# Application of extracorporeal membrane oxygenation in respiratory and circulatory support of preterm infants: a multicenter retrospective study in China

**DOI:** 10.3389/fcvm.2026.1885674

**Published:** 2026-07-28

**Authors:** Jingke Cao, Feng Wang, Zhe Zhao, Feng Wang, Hanwu Huang, Zhangxue Hu, Yuxiong Guo, Guoping Lu, Rong Ju, Zhiwei Zhang, Xiaoyang Hong, Qiuping Li

**Affiliations:** 1 The Second School of Clinical Medicine, Southern Medical University, Guangzhou, China; 2 Department of Neonatology, Senior Department of Pediatrics, The Seventh Medical Center of PLA General Hospital, Beijing, China; 3 Pediatric Intensive Care Unit, Senior Department of Pediatrics, The Seventh Medical Center of PLA General Hospital, Beijing, China; 4 Surgical Care Unit, Henan Children’s Hospital, Children’s Hospital Affiliated to Zhengzhou University, Zhengzhou, China; 5 Pediatric Intensive Care Unit, The People's Hospital of Guangxi Zhuang Autonomous Region, Nanning, China; 6 Department of Pediatrics, Daping Hospital, Third Military Medical University (Army Medical University), Chongqing, China; 7 Pediatric Intensive Care Unit, Department of Pediatrics, Guangdong Provincial People's Hospital, Guangdong Academy of Medical Sciences, Guangzhou, China; 8 Pediatric Intensive Care Unit, Children's Hospital of Fudan University, Shanghai, China; 9 Department of Neonatology, School of Medicine, Chengdu Women's and Children's Central Hospital, University of Electronic Science and Technology of China, Chengdu, China

**Keywords:** brain injury, extracorporeal membrane oxygenation (ECMO), preterm infant, prognosis, veno-arterial ECMO

## Abstract

**Objective:**

Extracorporeal membrane oxygenation (ECMO) serves as a vital rescue therapy for neonates with refractory cardiopulmonary failure, yet domestic clinical data regarding ECMO use in preterm infants remain scarce. This study sought to summarize clinical experiences and long-term prognostic outcomes of ECMO support among preterm infants in China.

**Methods:**

We conducted a retrospective analysis of preterm infants receiving ECMO between January 2018 and November 2025 using data from the Pediatric Extracorporeal Life Support Alliance of China (PELSAC). The primary study outcomes were ECMO weaning success and survival to hospital discharge. Secondary outcomes included ECMO-associated complications, intracranial hemorrhage and hypoxic-ischemic brain injury detected by cranial imaging, and neurodevelopmental delay assessed at corrected age 18–24 months. Clinical outcomes were compared between infants with birth gestational age ≤34 weeks and those >34 weeks.

**Results:**

Six out of 23 participating PELSAC centers (26.1%) implemented ECMO for 22 preterm infants, including 8 infants with gestational age ≤34 weeks and 14 infants over 34 weeks. The cohort had a median gestational age of 34 (32–36) weeks, a median birth weight of 2.5 (1.80–3.63) kg, and a median cannulation weight of 2.2 (1.80–2.70) kg. The median ECMO runtime was 112 (19–245) hours. 18 infants (81.8%) were successfully weaned, and 17 (77.3%) survived discharge. Complications were limited to one cannulation-site bleed and one thrombosis. Post-ECMO MRI detected brain injury in 9 of 17 survivors (52.9%), including 5 intracranial hemorrhages (29.4%) and 4 isolated hypoxic–ischemic lesions. At 18–24 months corrected age, 10 survivors (58.8%) had normal neurodevelopment, while 7 (41.2%) had developmental delay. Outcomes did not differ significantly between the ≤34-week and >34-week subgroups.

**Conclusion:**

ECMO in preterm infants (≥32 weeks’ gestation, ≥1.8 kg) achieves a high survival rate, although neurological complications remain common and require close surveillance. These findings support the potential for wider clinical application of ECMO in carefully selected preterm infants.

## 1 Introduction

As a critical cardiopulmonary replacement technology, extracorporeal membrane oxygenation (ECMO) can partially or completely replace cardiopulmonary function to maintain systemic oxygenation, and has become an important method for treating critically ill neonates unresponsive to conventional treatment (1, 2). According to the report of the Extracorporeal Life Support Organization (ELSO) (3), neonates who received ECMO accounted for about 29.8% of all worldwide registered ECMO cases in 2020, offering a hospital discharge survival rate of 65.3%, indicating that ECMO has significant value in the treatment of neonatal critical illnesses such as severe respiratory distress syndrome and persistent pulmonary hypertension of the newborn.

However, the application of ECMO in the more vulnerable preterm infant population has progressed slowly (4, 5). Preterm infants have immature organ development, especially those with birth gestational age <34 weeks and birth weight ≤2 kg. Not only is it difficult to establish vascular access due to their delicate blood vessels, but they also have an immature coagulation mechanism, which is prone to serious complications such as intracranial hemorrhage (ICH). Therefore, preterm infants were once considered a relative contraindication for ECMO application (6, 7). In addition, the types and progression of diseases in preterm infants are unique. Most existing ECMO indication and contraindication guidelines are only applicable to term infants, and also lack targeted standards. There is also no mature consensus on anticoagulation and coagulation management as well as prevention and control of neurological injury. These factors have jointly restricted the clinical application of ECMO in preterm infants (8).

Compared with the United States and European countries, where neonatal ECMO was first implemented clinically in 1975 and numerous registry-based retrospective cohort studies have been released over the past decades (9, 10), neonatal ECMO practice in China began far later, particularly for preterm infants. Only limited single-center case reports have been published, and systematic multi-center analyses are still lacking (8, 11). Optimized ECMO hardware and refined clinical techniques have substantially improved treatment outcomes for low-gestational-age and low-birth-weight preterm infants, highlighting an urgent need to reassess traditional contraindication criteria. Against this backdrop, the present study aimed to summarize clinical experience and prognostic outcomes of preterm infants receiving ECMO support via analysis of clinical data from the Pediatric Extracorporeal Life Support Alliance of China (PELSAC) database collected from January 2016 to November 2025. Our findings aim to furnish clinical evidence to broaden ECMO use in Chinese preterm infants and refine treatment protocols, ultimately improving preterm survival and lowering related mortality and morbidity.

## 2 Materials and methods

### 2.1.1 study subjects

Clinical data of preterm infants who received ECMO support from the database of the PELSAC between January 2018 and November 2025 were retrospectively analyzed. The inclusion criteria were preterm infants with birth gestational age <37 weeks who received ECMO support due to cardiopulmonary failure unresponsive to conventional treatment. Infants younger than 32 weeks' gestation were not considered for ECMO in this study. Patients were considered for ECMO support if they met any of the following criteria (11): (1) Oxygenation Index (OI) > 40 for more than 4 h, or OI >20 for more than 24 h; (2) Severe hypoxic respiratory failure with acute decompensation (arterial partial pressure of oxygen PaO₂ < 40 mmHg) unresponsive to maximum medical treatment (including high-frequency ventilation and inhaled nitric oxide); (3) Severe pulmonary hypertension with evidence of right ventricular dysfunction and/or left ventricular dysfunction; and (4) Shock and refractory hypotension with evidence of end-organ hypoperfusion despite maximum medical treatment. Strict patient selection was applied in this study, including routine daily cranial ultrasound during ECMO for monitoring intraventricular hemorrhage (IVH), pre-cannulation exclusion of infants with Papile grade III–IV IVH, restriction to infants with a gestational age of ≥32 weeks and body weight of ≥1.8 kg, and exclusion of those with fatal congenital anomalies or irreversible brain injury; these criteria were adopted to minimize neurological risk.

Definition and classification of primary diagnosis: Primary disease was defined as the fundamental pathological disorder that led to refractory cardiopulmonary failure requiring ECMO support. For patients presenting with pulmonary hypertension: Idiopathic severe persistent pulmonary hypertension of the newborn (PPHN) without identifiable underlying pulmonary disease, congenital cardiac malformation, sepsis, or severe asphyxia was classified as the primary diagnosis; When PPHN developed secondary to respiratory distress syndrome, air leak, congenital lung malformation, sepsis, or severe perinatal asphyxia, the underlying primary lung/infectious/hypoxic disorder was recorded as the main diagnosis, and PPHN was listed as a secondary hemodynamic complication; For infants with structural congenital heart disease combined with pulmonary hypertension, cardiac malformation was regarded as the primary disease.

### 2.2 Study methods

#### 2.2.1 ECMO support protocol

##### 2.2.1.1 Mode selection

Most preterm infants required simultaneous cardiac and respiratory support upon admission; therefore, all infants were supported with veno-arterial (VA) ECMO.

##### 2.2.1.2 Cannulation method

Referring to the standard procedure for cervical vessel cannulation, puncture and cannulation were performed through the right common carotid artery and internal jugular vein. Arterial cannulation was performed using ligation or vessel-sparing technique, and venous cannulation was performed using ligation technique. Cannulas of 8–10 Fr size were selected according to the infant's weight. At decannulation, the common carotid artery was ligated in all patients; no arterial repair was attempted. This approach followed standardized protocols of all included centers, given the infants' tiny fragile carotid vessels raising repair-related bleeding, thrombosis and stenosis risks, limited institutional experience with neonatal carotid microsurgery, and the priority to avert acute perioperative complications despite potential long-term cerebrovascular hazards from ligation.

##### 2.2.1.3 Parameter setting

A centrifugal pump was used to provide circulatory power, by maintaining the pump flow rate at 80–120 mL/(kg·min) and a target mixed venous oxygen saturation of 70%–80%. Continuous heparin anticoagulation therapy was performed during ECMO support to maintain an activated clotting time (ACT) of 180–220 s. Anti-Xa activity and activated partial thromboplastin time (aPTT) were not routinely monitored in this multicenter retrospective cohort. The ventilator was adjusted to ‘rest mode’ with a peak inspiratory pressure of 15–22 cmH₂O, positive end-expiratory pressure of 5–8 cmH₂O, respiratory rate of 10–20 breaths/min, and fractional inspired oxygen concentration of 0.21–0.3.

##### 2.2.1.4 Transfusion thresholds

All infants receiving ECMO were managed with standardized transfusion parameters targeting a hemoglobin level of at least 120 g/L for red blood cells and a platelet count of at least 100 × 10⁹/L, with no separate transfusion thresholds used for preterm infants. A target fibrinogen concentration of ≥1.5 g/L was maintained throughout ECMO support. Fresh frozen plasma was transfused for fibrinogen levels between 1.0 g/L and 1.5 g/L accompanied by mild active bleeding, while cryoprecipitate was given when fibrinogen was <1.0 g/L or complicated with severe hemorrhage including pulmonary hemorrhage and ICH.

##### 2.2.1.5 Fluid balance and hemodynamic management

Daily fluid balance was closely monitored throughout ECMO support. Total fluid intake was restricted as appropriate, and diuretics were administered to infants with tissue edema, pulmonary congestion or elevated central venous pressure to prevent fluid overload. Vasoactive agents (inotropes and vasopressors) were individually titrated based on real-time blood pressure, peripheral perfusion, serum lactate and urine output. Medication types and doses were dynamically adjusted to sustain stable systemic perfusion and avoid excessive vasoconstriction that may compromise cerebral and visceral perfusion.

##### 2.2.1.6 ECMO weaning criteria

ECMO weaning criteria were formulated with reference to the pediatric circulatory support guidelines released by the ELSO (12).

#### 2.2.2 Data collection and follow-up observation

Baseline data of infants were collected, including birth gestational age, birth weight, gender, weight at cannulation, and ECMO Indication; ECMO-related data, including support mode, cannulation method, support duration, and anticoagulation management parameters; clinical outcome data, including weaning success rate, hospital survival rate, and incidence of complications. After weaning, cranial MRI was performed on surviving infants to assess brain injury, and neurodevelopmental status was evaluated through outpatient follow-up or telephone follow-up in the later period.

Neuroimaging: Cranial ultrasound was performed daily during ECMO to screen for IVH and acute brain injury. After weaning, survivors underwent cranial MRI to characterize brain injury (hemorrhage, hypoxic–ischemic lesion, stroke, severity). Neurodevelopment was assessed at corrected age 18–24 months using the Bayley Scales of Infant and Toddler Development via outpatient follow-up or telephone contact.

#### 2.2.3 Study outcomes

Primary outcomes included successful ECMO weaning (sustained stable cardiopulmonary function after tapering ECMO flow without re-initiation) and survival to hospital discharge. Secondary outcomes covered ECMO-related complications (cannulation bleeding, thrombosis, acute kidney injury), post-ECMO ICH and hypoxic–ischemic brain injury confirmed by cranial MRI in survivors, and neurodevelopmental delay evaluated by the Bayley Scales at corrected age 18–24 months.

## 3 Statistical analysis

SPSS 25.0 software was used for data processing. Measurement data are expressed as median (minimum-maximum); count data are expressed as number of cases (constituent ratio, %), and inter-group comparison was performed using the *χ^2^* test. When the theoretical frequency was <5, Fisher's exact test was used. *P* < 0.05 was considered statistically significant.

## 4 Results

### 4.1 General situation

A total of 22 preterm infants received ECMO support during the study period. Patient demographics and clinical data are presented in Table 1. The cohort included 15 males (68.2%) and 7 females (31.8%), among whom 8 infants had a gestational age ≤34 weeks, with the minimum gestational age of 32 weeks. The median birth weight was 2.5 (1.80–3.63) kg. All 22 infants were managed with VA ECMO, with a median ECMO runtime of 112 (19–245) hours. Leading clinical indications for ECMO support were severe PPHN (11, 50%) and sepsis (3, 13.6%). 18 infants (81.8%) achieved successful ECMO weaning, and 17 (77.3%) survived to hospital discharge; 5 infants died, including 1 due to ECMO failure and 4 after withdrawal of life-sustaining treatment. Only 1 patient developed cannulation-site bleeding and 1 experienced vascular thrombosis during ECMO, while no cases of acute kidney injury were observed.

**Table 1:** Baseline characteristics and clinical outcomes of preterm infants receiving ECMO support.

Item	Number of cases (n, %) or median (minimum-maximum)
Birth Gestational Age	34 weeks (29–36 weeks)
Birth Weight	2.50 kg (1.80–3.63 kg)
Gender (Male/Female)	15/7
Delivery Mode (Cesarean Section)	16 (72.7)
1-minute Apgar Score	7 (0–10)
5-minute Apgar Score	6 (0–10)
10-minute Apgar Score	6 (0–10)
ECMO Indication	
Severe PPHN	11 (50)
Sepsis	3 (13.6)
Severe Asphyxia with HIE	2 (9.1)
Severe Air Leak	2 (9.1)
Congenital Diaphragmatic Hernia	1 (4.5)
Coarctation of the Aorta	1 (4.5)
Congenital Cystic Adenomatoid Malformation of the Lung	1 (4.5)
Congenital Capillary Dysplasia Syndrome	1 (4.5)
ECMO runtime	112 (19–245)
ECMO Weaning Success Rate	18 (81.8)
Death	5 (22.7)
Recovery and Discharge	17 (77.3)

ECMO, extracorporeal membrane oxygenation; PPHN, persistent pulmonary hypertension of the newborn; HIE, hypoxic–ischemic encephalopathy.

### 4.2 Outcomes of ECMO support in preterm infants with different gestational Age

Numerically, infants with gestational age ≤34 weeks exhibited slightly lower rates of successful ECMO weaning and hospital survival, alongside marginally higher incidences of ICH and neurodevelopmental delay relative to those born after 34 weeks (Table 2). Neurological outcome indicators exclude one survivor with baseline hypoxic–ischemic encephalopathy (HIE) to remove pre-existing brain injury confounding. The ECMO-related clinical information of all 8 preterm infants with birth gestational age ≤34 weeks is shown in Table 3. The minimum weight at cannulation was 1.8 kg, the minimum gestational age was 32 weeks, the median ECMO runtime was 131 h, and finally 2 infants died after withdrawal from treatment due to PPHN in one and mediastinal emphysema in the other.

**Table 2:** Outcomes of ECMO support in preterm infants with different gestational ages.

Group	N(%)	ECMO runtime(h)	ECMO Weaning Success (n, %)	In-hospital Death (n, %)	Survival to Discharge (n, %)	Intracranial Hemorrhage (n, %)	Hypoxic Brain Injury (n, %)	Neurodevelopmental Delay(n, %)
>34 weeks	14 (63.6)	119 (19–245)	12 (85.7)	3 (21.4)	11 (78.6)	3 (27.3)	1 (10.0)^a^	4 (40.0)^a^
≤34 weeks	8 (36.4)	131 (103–236)	6 (75.0)	2 (22.2)	6 (75.0)	2 (33.3)	1 (16.6)	3 (50.0)
*P* value		NA	0.72	1	1	1	1	1

The extremely small sample size results in severely insufficient statistical power. All *P*-values are only for exploratory data description and cannot support statistical inference or clinical generalization.

ECMO, extracorporeal membrane oxygenation.

a One survivor with baseline hypoxic–ischemic encephalopathy was excluded from analyses of hypoxic brain injury and neurodevelopmental delay to avoid confounding bias. All cases of hypoxic brain injury presented in the table represent new lesions acquired after ECMO initiation and do not include cerebral lesions caused by pre-existing HIE.

**Table 3:** Clinical data of infants with birth gestational Age ≤34 weeks.

No.	GA (weeks)	Sex	Cannulation weight (kg)	Diagnosis	ECMO mode	Vessel cannulation	Catheter size	ECMO runtime (h)	Hospital outcome	ICH	Hypoxic brain injury	Vascular thrombosis	Neurodevelopmental delay
1	32	F	1.82	PPHN	VA	CCA + IJV	8Fr A/10Fr V	103	Death after withdrawal	-	No	No	NA
2	34	F	2.32	Mediastinal emphysema	VA	CCA + IJV	8Fr A/10Fr V	113	Death after withdrawal	-	No	No	NA
3	34	F	1.90	PPHN	VA	CCA + IJV	8Fr A/10Fr V	112	Survived discharge	No	No	No	Yes
4	34	M	2.70	Congenital cystic adenomatoid malformation	VA	CCA + IJV	8Fr A/10Fr V	142	Survived discharge	No	No	No	Yes
5	34	M	2.46	PPHN	VA	CCA + IJV	8Fr A/10Fr V	233	Survived discharge	No	Yes	No	No
6	34	M	2.00	Congenital diaphragmatic hernia	VA	CCA + IJV	8Fr A/10Fr V	169	Survived discharge	Yes	No	Yes	No
7	34	M	2.55	PPHN	VA	CCA + IJV	8Fr A/10Fr V	236	Survived discharge	No	No	None	No
8	33	F	1.80	PPHN	VA	CCA + IJV	8Fr A/10Fr V	120	Survived discharge	Yes	No	None	Yes

GA, gestational age; ECMO, extracorporeal membrane oxygenation; ICH, intracranial hemorrhage; F, female; M, male; PPHN, persistent pulmonary hypertension of the newborn; VA, veno-arterial; CCA, common carotid artery; IJV, internal jugular vein; A, arterial; V, venous; ICH, intracranial hemorrhage; —, absent; NA, not assessed (deceased before follow-up).

## 5 Discussion

Based on multi-center data from the PELSAC, this study is the first to systematically report the clinical experience and prognosis of ECMO application in preterm infants in China. Although the sample size is limited, this study reveals that under strict screening and management, preterm infants can achieve encouraging survival rates with ECMO, and also clarifies that neurological complications remain a key concern.

The successful ECMO weaning rate in our cohort of premature infants is 81.8% (18/22), and the hospital discharge survival rate is 72.7% (16/22), which is close to the 76% hospital discharge survival rate reported by Schueller et al. (13). This positive prognosis is mainly due to the combined effect of ECMO technological advancement and precise patient selection. Technological advancement is reflected in the popularization of smaller-sized ECMO consumables (suitable for low-weight infants), precision centrifugal pumps, and bedside imaging monitoring technologies such as echocardiography and near-infrared spectroscopy after 2018, which lays a solid foundation for refined management. Meanwhile, all cases in our series were from medical centers with mature pediatric extracorporeal life support qualifications, and the experience of centralized expert teams and standardized processes further ensured the treatment effect. The strict inclusion criteria (excluding grade III and above IVH) selected the group most likely to benefit from ECMO treatment. The primary diseases treated with ECMO are also directly related to the prognosis. Previous studies have shown that the mortality rate of congenital diaphragmatic hernia (CDH) is higher than that of respiratory failure and PPHN (11). In this study, there was only one case of CDH, and most were infants with PPHN and respiratory failure, which may also be the reason for the higher survival rate. A recent systematic review covering 36 studies further confirms the value of ECMO technological advancement (14). Data show that with technological progress and management optimization, the incidence of ICH has decreased from 89% to 21%, and the survival rate of ECMO treatment in early preterm infants has increased from 25% to 76% (similar to that of late preterm infants). Therefore, some researchers challenge the standard in the ELSO guidelines that ‘gestational age <34 weeks is a relative contraindication for ECMO’ and recommend the establishment of an international working group to revise the existing guidelines.

Preterm infants, especially those with gestational age <34 weeks, have long been considered a relative contraindication for ECMO due to their delicate blood vessels, immature coagulation function, and high risk of ICH (15). However, with the iteration of medical technology, this concept is being re-evaluated (16). As evidenced by early investigations including Bartlett's work, preterm infants undergoing ECMO faced a daunting 89% risk of developing ICH (15), in sharp contrast, recent data from Olutoye et al. (17) have shown that this incidence has been significantly reduced to 38%, reflecting substantial progress in clinical management. In our study, the overall incidence of ICH is 29.4% (5/17), and the incidence of ICH in the ≤34-week group is 33.3% (2/6), which supports the overall decreasing trend of ECMO-associated complications, marking an important paradigm transition of the core challenge of ECMO in preterm infants from the initial ‘technical feasibility’ and ’survival possibility’ to how to maximize the prevention and management of neurological complications, especially ICH, under the precondition of ensuring survival.

All cases in this group were supported with the VA mode, and 20 of the 22 premature infants were for respiratory support. Although the VA mode may theoretically increase the risk of cerebral hemodynamic instability and bleeding (18), most preterm infants in this study did not develop ECMO - associated ICH. The key management strategies include: ① Conservative anticoagulation (ACT 180–220 s) adapted to the preterm infant's coagulation system; ② Standardized vascular cannulation to reduce injury; ③ Close neuroimaging monitoring (all survivors underwent cranial MRI) after treatment and weaning. The key findings of our study are as follows: The incidence of ICH in the ≤34 weeks group was slightly higher, but the hospital discharge survival rate of 75% was comparable to 78.6% in the >34 weeks group, suggesting that after strict evaluation and refined management, the survival benefit of VA ECMO may outweigh the potential bleeding risk, though this conclusion needs to be verified by large-sample prospective studies.

The neurodevelopmental outcome is the gold standard for evaluating the long-term value of ECMO in preterm infants. We found a ‘disconnection between injury and function’ phenomenon In our study, showing that 8 (47.1%) of the 17 ECMO survivors presented with brain injury on cranial MR imaging, and 7 (41.2%) with neurodevelopmental delay. Notably, among the 8 preterm infants with gestational age ≤34 weeks, 3 had neurodevelopmental delay after ECMO support, but only 1 of them had ICH, suggesting that the etiology of neurodevelopmental delay is likely complex and multifactorial rather than attributable to ICH alone. These findings are consistent with those in the term neonates receiving ECMO in our center (56% of the 50 survivors had abnormalities on cranial MR imaging), and the long-term follow-up results of Madderom et al. (19) who reported. This phenomenon is largely due to the plasticity of the developing brain, and multi-factorial influences on neurodevelopment such as genetics/family environment/early intervention, while the current evaluation of ‘neurodevelopmental delay’ may not be able to identify subtle cognitive/behavioral problems. Therefore, it is necessary to establish a long-term systematic follow-up mechanism in ECMO survivors of preterm infants based on standardized tools (such as the Bayley scale), knowing that they are no less important than acute-phase life support.

This study has some limitations: ① As a retrospective multi-center study, there are differences in patient selection thresholds, anticoagulation management, and neurodevelopmental follow-up protocols between different centers, which may introduce bias; ②The overall sample size is limited, with only 8 patients in the subgroup of gestational age ≤34 weeks. This study is underpowered for formal inferential statistics, and all intergroup comparison results should be interpreted for descriptive and exploratory purposes only.; ③ Anti-Xa/aPTT not uniformly monitored. ④Standardized electrophysiological monitoring including aEEG or cEEG was not universally implemented at participating centers; subclinical seizures could not be systematically screened, and neurological assessment relied only on structural cranial imaging. Some infants had short neurodevelopmental follow-up time and inconsistent evaluation methods, making it impossible to evaluate executive function, learning, and behavioral problems in childhood.

## 6 Conclusion

Nevertheless, ECMO is effective and may confer an acceptable safety profile in carefully selected preterm infants (≥32 weeks gestation, ≥1.8 kg weight) with refractory cardiopulmonary failure. Neurological complications remain frequent, necessitating rigorous cerebral imaging and long-term neurodevelopmental follow-up. Larger prospective cohorts, unified clinical protocols, and national long-term follow-up networks are required to optimize ECMO indications, anticoagulation strategies, and long-term patient outcomes.

## Data Availability

The raw data supporting the conclusions of this article will be made available upon reasonable request to the corresponding author, in compliance with ethical regulations and data protection policies.
